# Screening of Rosemary Essential Oils with Different Phytochemicals for Antioxidant Capacity, Keratinocyte Cytotoxicity, and Anti-Proliferative Activity

**DOI:** 10.3390/molecules28020586

**Published:** 2023-01-06

**Authors:** Yeqin Huang, Heran Xu, Mengting Ding, Jingyi Li, Di Wang, Hui Li, Meiyu Sun, Fei Xia, Hongtong Bai, Min Wang, Meiling Mo, Lei Shi

**Affiliations:** 1 Key Laboratory of Plant Resources and Beijing Botanical Garden, Institute of Botany, Chinese Academy of Sciences, Beijing 100093, China; 2 China National Botanical Garden, Beijing 100093, China; 3 College of Life Sciences, University of Chinese Academy of Sciences, Beijing 100049, China; 4 Beijing Key Laboratory of Plant Research and Development, College of Chemistry and Materials Engineering of Beijing Technology and Business University, Beijing 100048, China; 5 Sinno Cosmetics Co., Ltd., Zhongshan 528451, China

**Keywords:** rosemary essential oils, chemical composition, antioxidant, cytotoxicity, cancer, α-pinene

## Abstract

Nowadays, the demand for rosemary essential oils (REOs) in the cosmetic, food, and pharmaceutical industries is increasing, and the abundant germplasm resources of rosemary provide more possibilities for functional applications. The REOs from six cultivars were selected to evaluate and compare their bioactivities. REOs have good cellular antioxidant activity in scavenging reactive oxygen species, and the technology for order preference by similarity to an ideal solution (TOPSIS)-random forest multivariate model indicated that ‘Dutch Mill’ REO has the best antioxidant activity, which is closely related to its verbenone content. In addition, α-pinene-dominant REOs are more toxic to human keratinocytes, which is closely related to the content of α-pinene, as revealed by multivariate analyses. Moreover, anti-proliferative assays on six cancer cell lines showed that all REOs have a higher anti-proliferative ability against human pancreatic cancer cell line SW1990 and gastric epithelial cell line NCI-N87. Among them, ‘Miss Jessopp’s Upright’ and ‘Blue Lagoon’ REOs exhibit more prominent anti-proliferative activity. Our study provides a reference value for exploring the application potential of different REOs by evaluating their differences in chemical composition and bioactivity.

## 1. Introduction

*Rosmarinus officinalis* L. (Labiaceae), commonly known as rosemary, is an aromatic plant native to the Mediterranean region. Rosemary essential oils (REOs) have been widely used in perfumery, cooking, and disease treatment since ancient times [1] and are recognized in many countries and documented in the official pharmacopeia [2]. With the recognition of their antibacterial, insecticidal, anti-inflammatory, and antioxidant activities [3], they have been extensively applied in disinfection, cosmetics, and aromatherapy [4]. In addition, these promising natural products have been innovatively applied in other fields as non-antibiotic feed additives [5], new packaging systems [6], and drug delivery systems [7], among others [8].

Plants have different chemotypes as genetic factors and environmental conditions cause permanent changes in the chemical composition of their essential oils [9]. The major components of REOs are proven to be α-pinene, 1,8-cineole, camphor, verbenone, borneol, and limonene [10]. Several common chemotypes of REOs have been reported and are usually named after one or two major components, such as α-pinene, camphor, 1,8-cineole, and verbenone [11,12]. Each chemotype comprises different composition types and contents, thus exhibiting different activities [13,14]. The Chinese National Germplasm Bank of Aromatic Plants has collected many rosemary cultivars from around the world, which prompted us to evaluate the chemical composition and activity of different REOs.

The antioxidant activity of essential oils is closely related to the chemical structure of terpenoids, such as the benzene ring linking to hydroxyl groups and the double bonds, providing a greater ability to eliminate free radicals [15]. In addition to the common chemical assays, such as 1,1-Diphenyl-2-(2,4,6-trinitrophenyl)diazen-1-ium (DPPH) and 2,2’-Azinobis-(3-ethylbenzthiazoline-6-sulphonate (ABTS) assays, free radical scavenging experiments at the cellular level can more authentically reflect the effects of natural products on human health. The differences in the reactive oxygen species (ROS) scavenging ability among REOs were demonstrated in this study.

REOs are important ingredients used in cosmetic formulations. The growing consumer awareness of their origin and safety has led us to test for safer raw materials. Cell viability assays are widely used to measure cellular responses to toxic agents in drug screening [16]. Thus, the cytotoxicity and effect of REOs on the viability of keratinocytes (HaCaT) were tested.

Among the various diseases known all over the world, cancer is the major cause of death. Traditional synthetic medicines, although very effective when used, can also have many side effects. As a new, safer alternative, essential oils offer multiple benefits in cancer therapy through targeted drug delivery systems using ligands, protein microspheres, or nanomaterials. Ongoing clinical studies have indicated that REOs have promising pharmacological applications [17]. Therefore, it is necessary to explore the anticancer potential of different REOs against different cancer cells.

Based on six selected rosemary cultivars, this study focused on the chemical properties of REOs and assessed their differences in antioxidant activity, keratinocyte cytotoxicity, and the inhibition of cancer cell proliferation. In addition, we used multiple multivariate analysis methods to explore the relationship between components and activities and select cultivars with different value advantages, which pointed out the direction of breeding on the one hand and provided clues for further exploration and verification of the function of natural products on the other hand.

## 2. Results

### 2.1. Chemical Profiling

**Figure 1:**
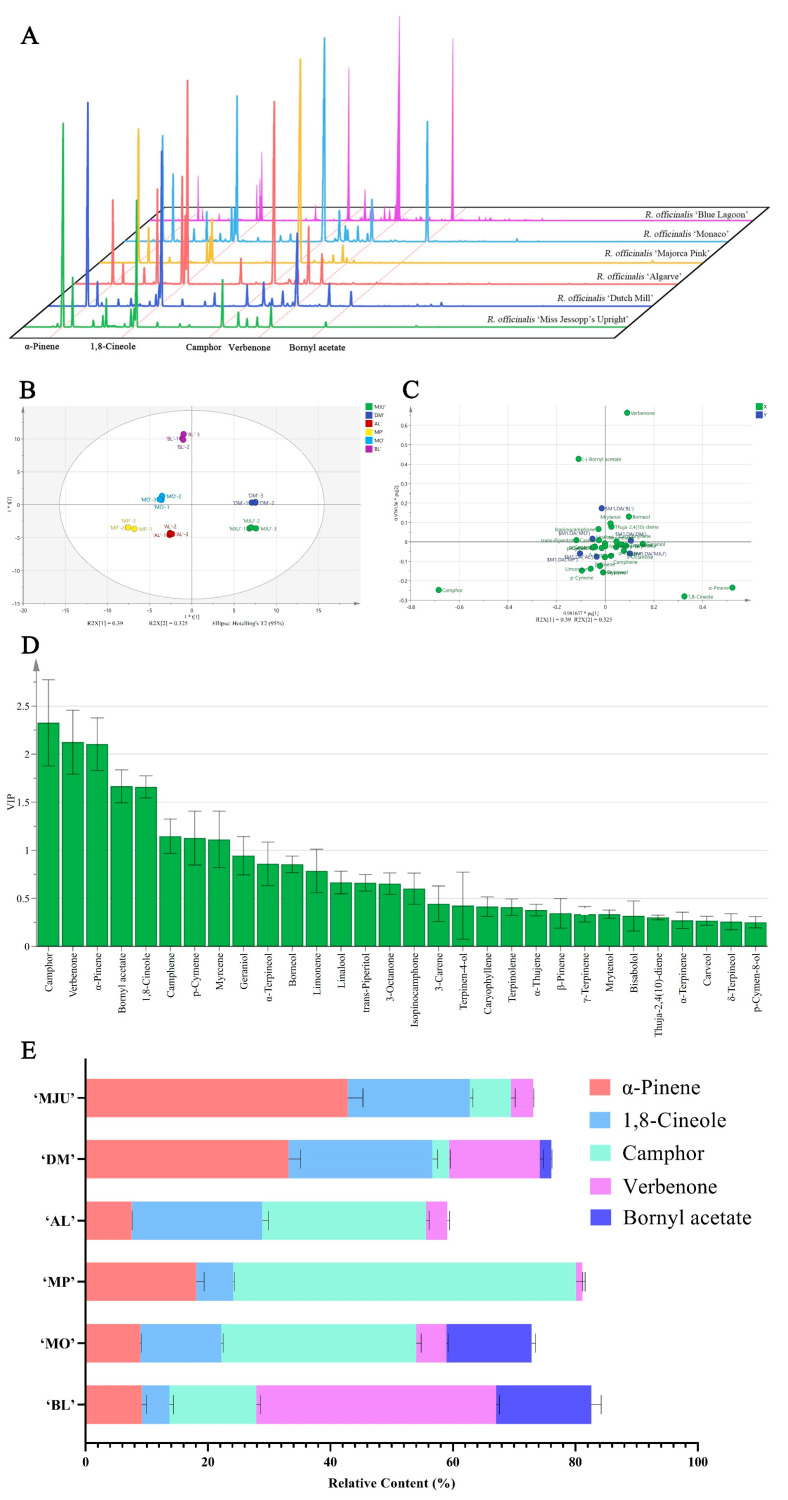
The analysis of chemical composition difference of REOs. (**A**) The chromatograms. (**B**) The scores plot summarizing the relationship among the REOs in the OPLS-DA model. (**C**) The loadings plot summarizing the relationship among the constituents in the OPLS-DA model. (**D**) The VIP plot summarizing the importance of the EO constituents both to explain X and to correlate Y in the OPLS-DA model. (**E**) The main chemical composition of REOs.

GC-MS analysis identified 30 components in REOs from six cultivars (Figure 1A, Table 1). Among them, monoterpenes and oxygenated monoterpenes accounted for 98.41–100%. Orthogonal partial least squares-discriminant analysis (OPLS-DA) is a suitable multivariate method for analyzing massive and complex chromatographic data because it can enhance relevant information and decrease unrelated noises. Hence, we built an OPLS-DA model based on the chemical profiling of REOs (Figure 1B,C), where the VIP value reflects the importance of each component in REOs. A VIP value > 1 is an indicator that could best explain the difference (Figure 1D). The results revealed that camphor, verbenone, α-pinene, bornyl acetate, 1,8-cineole, camphene, p-cymene, and myrcene contribute the most to distinguishing the differences among the six REOs. Camphor, verbenone, α-pinene, 1,8-cineole, and bornyl acetate are the five most important variates (VIP > 1.5), accounting for 59.08–81.12% by mass of these REOs with different chemical compositions (Figure 1E). The α-pinene content is 42.81 ± 2.52% in the REO from ‘Miss Jessopp’s Upright’ (‘MJU’) and 33.15 ± 1.98% in the REO from ‘Ducth Mill’ (‘DM’). Camphor is the main component in REOs from ‘Majorca Pink’ (‘MP’), ‘Monacao’ (‘MO’), and ‘Algarve’(‘AL’), accounting for 56.04 ± 1.47%, 31.86 ± 0.82%, and 26.80 ± 0.51%, respectively. Verbenone is the most abundant component in ‘BL’ REO, accounting for 39.14 ± 0.55%.

### 2.2. Antioxidant Activity

**Figure 2:**
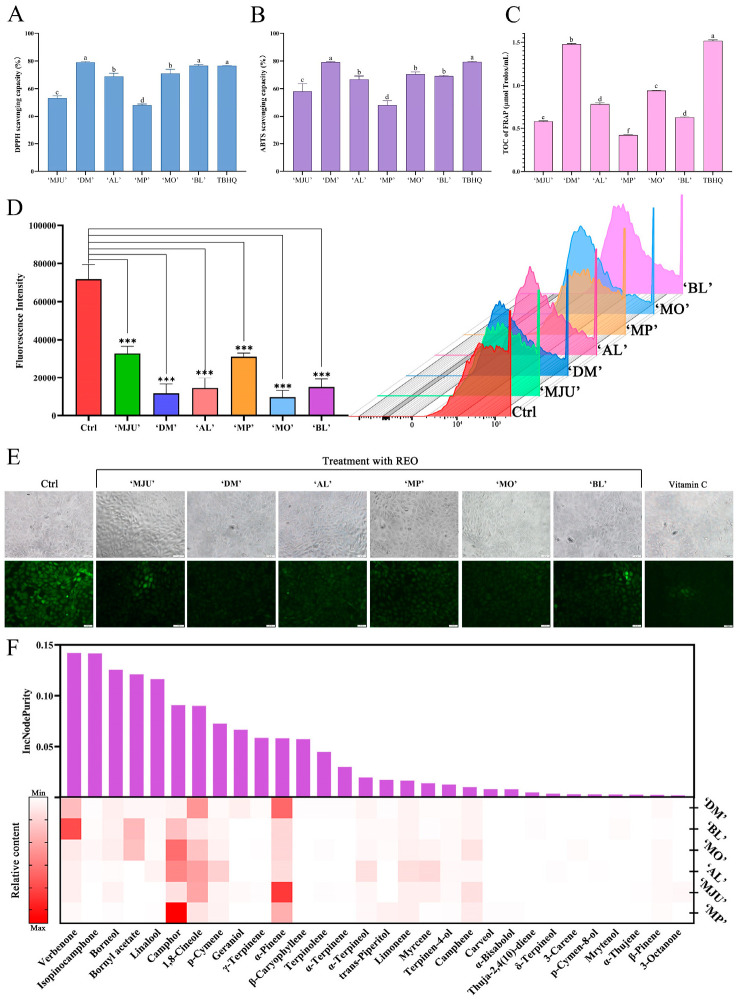
The antioxidant capacity of REOs by different assays. (**A**) DPPH assay. (**B**) ABTS assay. (**C**) FRAP assay. (**D**) The scavenging capacity of ROS in HaCaT. (**E**) The change of ROS intensity under fluorescence microscope. (**F**) Random forest regression analysis model based on components and the overall antioxidant activity of TOPSIS. Data were expressed as means ± standard deviation (SD) (*** *p* < 0.001). Means with different letters in the same column are significantly different (*p* < 0.05).

To estimate the differences in the total antioxidant activity (TOC) of REOs from different cultivars in vitro, we used three chemical reaction assays. In the DPPH free radical scavenging test (Figure 2A), ‘DM’ and ‘BL’ REOs show the scavenging ratio as high as 79.02 ± 1.80% and 77.62 ± 0.75%, respectively. Consistently, ‘DM’ and ‘BL’ REOs show strong ABTS radical scavenging ability of 79.10 ± 0.35% and 70.50 ± 1.31%, respectively, in the ABTS radical scavenging test (Figure 2B). In the FRAP detection system (Figure 2C), ‘DM’ REO also shows the strongest TOC of 1.47 ± 0.01 μmol Trolox/mL. By contrast, ‘MP’ REO shows the lowest TOC of only 0.42 μmol Trolox/mL.

In addition to the above chemical reaction assays, the REOs’ ability to scavenge intracellular ROS was also examined in HaCaT cells using fluorescent probe DCFH-DA to reflect their antioxidant activity at the cellular level (Figure 2D,E). Intracellular ROS is the basis of all sorts of free radicals. ROS levels higher than a certain range can cause oxidative damage to cells. Therefore, we treated HaCaT cells with 100 μg/mL REOs, which is known to not cause any damage to cell viability, for 24 h. The REOs’ ROS scavenging capacity was calculated by changes in the ROS levels compared with vehicle treatment. The results showed that all REO treatments significantly reduce intracellular ROS levels by 54.21–86.55%, and the scavenging ability is ‘MO’, ‘DM’, ‘AL’, ‘BL’, ‘MP’, and ‘MJU’ in order from the strongest to the weakest.

Using the above REOs’ antioxidant capacity as an index, we applied the TOPSIS method to better evaluate the REOs’ overall antioxidant capacity and used the Ci value to rank the samples (Table 2). The larger the Ci value, the better the overall antioxidant ability. The results revealed that REOs with diverse chemical compositions have significantly different antioxidant abilities, and the ‘DM’ REO has better antioxidant ability than other REOs.

Random forest ensemble learners have excellent performance in regression analysis and provide estimates of chemical components’ importance. Therefore, we established a random forest model based on the results of TOPSIS and component differences to screen important components related to REOs’ overall antioxidant activity. Variable importance in the random forest regression model was calculated by IncNodePurity to represent the impact of each variable on the heterogeneity of the observed values at each node of the classification tree. The greater the IncNodePurity of the variable, the greater the importance of the variable. In the random forest regression analysis model (Figure 2F), verbenone, one of the main components in REOs, shows the most significant effect on the overall antioxidant activity of REOs, and insopinocamphone, borneol, bornyl acetate, and linalool also contribute greatly to the overall antioxidant activity of REOs.

### 2.3. Keratinocyte Cytotoxicity to HaCaT

**Figure 3:**
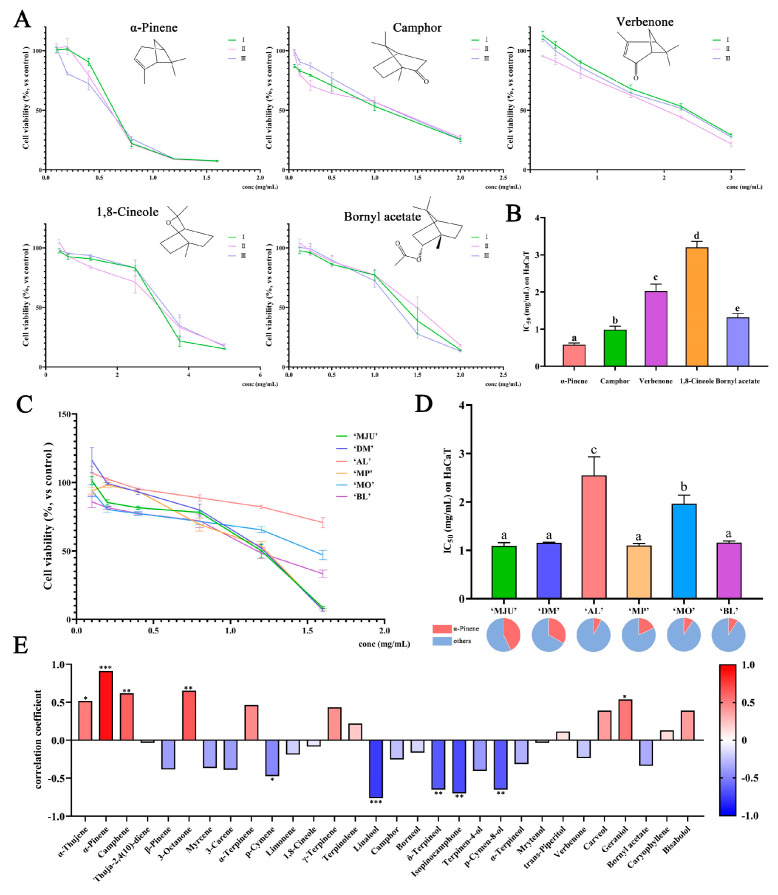
(**A**) The cytotoxicity of the 5 main compounds on HaCaT. (**B**) The IC_50_ of the 5 main compounds on HaCaT. (**C**) The cytotoxicity of REOs on HaCaT. (**D**) The IC_50_ of REOs on HaCaT. (**E**) The heatmap of Spearman correlation coefficient results between component and cytotoxicity. Data were expressed as means ± standard deviation (SD) (* 0.01 < *p* < 0.05, ** 0.001 < *p* < 0.01, *** *p* < 0.001). Means with different letters in the same column are significantly different (*p* < 0.05).

**Figure 4:**
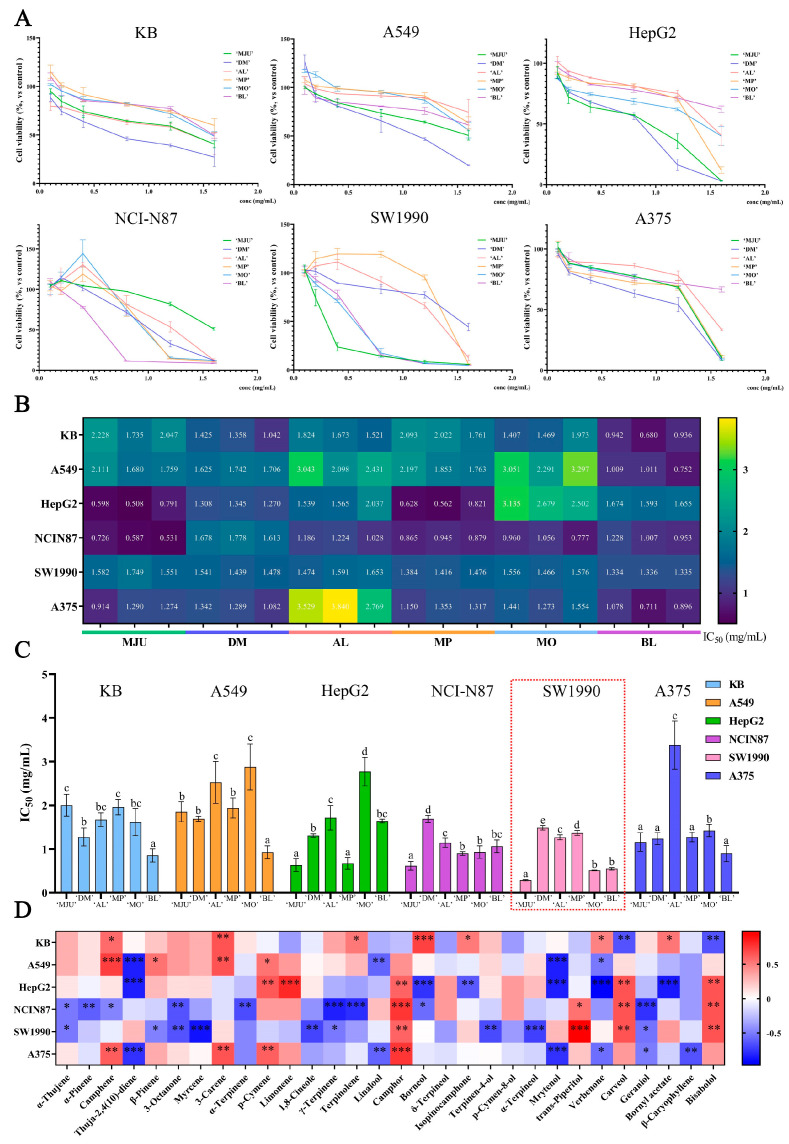
(**A**) The cytotoxicity of REOs on 6 cancer cells. (**B**) The heatmap of IC_50_ of REOs on 6 cancer cells. (**C**) The IC_50_ of REOs on 6 cancer cells. (**D**) The heatmap of Spearman correlation coefficient results between component and cytotoxicity. Data were expressed as means ± standard deviation (SD) (* 0.01 < *p* < 0.05, ** 0.001 < *p* < 0.01, *** *p* < 0.001). Means with different letters in the same cell line are significantly different (*p* < 0.05).

REOs have been widely used in cosmetics as skin care agents, food flavorings, and packaging systems. Thus, it is necessary to test the toxicity of REOs from different sources on skin cells. Keratinocytes account for about 95% of epidermal cells and are the most important cell type in human epidermal tissues [18]. In this study, human immortalized keratinocyte HaCaT was selected to evaluate the skin toxicity of REOs and their major components. We first tested the cytotoxicity of camphor, verbenone, α-pinene, bornyl acetate, and 1,8-cineole, the five main components of REOs, to reflect the toxicity trend of essential oil principal components (Figure 3A). The results showed that α-pinene is the most toxic, with an IC_50_ of 0.580 ± 0.039 mg/mL, followed by camphor (IC_50_ = 0.986 ± 0.075 mg/mL), Bornyl acetate (IC_50_ = 1.321 ± 0.080 mg/mL), Verbenone (IC_50_ = 2.021 ± 0.158 mg/mL), and 1,8-cineole (IC_50_ = 3.202 ± 0.130 mg/mL) (Figure 4B). Moreover, we also evaluated the cytotoxicity of the six REOs (Figure 3C). The results showed that the IC_50_ range of these REOs on HaCaT was 1.095–2.549 mg/mL (Figure 3D). Among the six REOs, ‘MJU’, ‘MP’, and ‘DM’ REOs are more cytotoxic, and ‘AL’ REO is the least cytotoxic. To elucidate the relationship between composition and cytotoxicity, we conducted a correlation matrix analysis (Figure 3E) and showed that α-pinene has the strongest association with cytotoxicity (r = 0.91, *p* < 0.001).

### 2.4. Anti-Proliferative Effect on Cancer Cells

The anti-proliferative ability of REOs was measured against six types of tumor cells involving the skin, pneogaster, and digestive system (Figure 4A). The results showed that the IC_50_ of these REOs against cancer cells varies from 0.285 to 3.379 mg/mL for 24 h of treatment (Figure 4B,C). Interestingly, these REOs show different inhibitory trends on different cancer cells, with the most prominent anti-proliferative activity against SW1990 and NCI-N87 cells. Moreover, REOs with different phytochemicals also show different anti-proliferative abilities. ‘MJU’ REO shows strong anti-proliferative activity against HepG2 (IC_50_ = 0.632 ± 0.118 mg/mL), NCI-N87 (IC_50_ = 0.615 ± 0.082 mg/mL), and SW1990 (IC_50_ = 0.285 ± 0.011 mg/mL) cells, but minimal anti-proliferative activity against KB cells (IC_50_ = 2.003 ± 0.204 mg/mL). Notably, ‘BL’ REO shows stronger anti-proliferative activity against KB, A549, and A375 cells than the other five REOs. In addition to ‘MJU’ and ‘BL’ REOs, ‘MP’ REO shows a strong ability to inhibit HepG2 cells, and ‘BL’ REO has evident anti-proliferative activity against SW1990 cells.

To identify the key components contributing to the difference in anti-proliferative activity, we analyzed the correlation of EROs’ IC_50_ with components (Figure 4D) using Spearman’s correlation matrix analysis. The results showed that camphor exhibits the highest correlation with abilities to inhibit multiple cells, including HepG2, NCI-N87, SW1990, and A375 cells. Monoterpene hydrocarbons camphene and 3-carene are significantly correlated with the anti-proliferative activity against KB, A549, and A375 cells, and monoterpene hydrocarbon p-cymene contributes significantly to the anti-proliferative activity against A549, HepG2, and A375 cells. In addition, borneol, one of the monoterpene alcohols, has the strongest positive association with toxicity to KB cells. Carveol and bisabolol are significantly associated with increased toxicity to HepG2 and NCI-N87 cells. Monoterpene alcohol *trans*-piperitol is closely associated with anti-proliferative activity against SW1990 cells.

## 3. Discussion

The study demonstrated significant differences in the composition of REOs from different rosemary cultivars, consistent with previous reports [19,20]. REOs are usually divided into several chemotypes, with the most common ones being α-pinene-type (α-pinene > 20%), 1,8-cineole-type (high content of 1,8-cineole), camphor-type (camphor > 20%), and verbenone-type (verbenone > 15%) [19,21]. Our selected REOs are well-represented in previously reported chemotypes. Therefore, it is of great indicative significance to evaluate their activity differences.

REOs have been proven to be effective antioxidants in vitro and in vivo [22,23,24,25]. In addition to the common assays to detect the differences in six REOs’ antioxidant capacities, we proved that REOs have a strong antioxidant ability to reduce ROS levels and inhibit free radical-mediated reactions, thereby protecting lipid and cellular components from oxidative stress [26,27]. More importantly, we comprehensively evaluated REOs’ bioactivity and explored the relationship between their antioxidant capacity and components using a TOPSIS-random forest multivariate analysis model. Although this model indicated that REOs’ antioxidant activity is strongly related to the content of some monoterpene ketones, it should be noted that the antioxidant properties of essential oils do not always directly depend on their main components and may be regulated by some of their minor components. Thus, the effects of synergy, antagonism, and additivity should be considered when linking the antioxidant properties of essential oils with their components [28].

Cytotoxicity is a key factor in many toxicological models because it involves the general toxicity mechanism shared by different cells [29]. This study selected a normal skin cell line to detect REOs’ cytotoxicity, intending to provide a safety reference for REOs from different plant sources in daily skincare applications and food packaging. Our results revealed that REOs rich in α-pinene, such as those from ‘MJU’ and ‘DM’, are more toxic to HaCaT, and multivariate analysis indicated that this toxicity is strongly related to their α-pinene content. Previous studies have demonstrated that α-pinene can induce cell death, possibly by modulating oxidative stress-related signaling pathways [30,31]. Hence, we should pay attention to the concentration and safety of REOs with high α-pinene content in daily skincare or food packaging.

In vitro cytotoxicity tests are of great importance and can be used as a screening method to identify potential anticancer drugs [32,33]. Wang et al. [34] have observed that REOs exhibited cytotoxic activities toward human cancer cells SK-OV-3, HO-8910, and Bel-7402. However, very few studies have been conducted on other cancer cells. Our inhibition assays on six cancer cells showed that REOs manifested more inhibiting potential in human pancreatic cancer cell line SW1990 and gastric epithelial cell line NCI-N87. Moreover, ‘MJU’ and ‘BL’ REOs have stronger anti-proliferation abilities than other REOs with different components. The IC_50_ below 1000 μg/mL is considered to be feasible for further anti-cancer activities [35,36,37], and the antiproliferation results enable us to further verify REOs with more targeted anticancer effects in vitro and in vivo. Our composition-activity relationship analysis showed that some important monoterpenes, including hydrocarbons and alcohols, contribute greatly to the REOs’ antiproliferation ability to multiple cancer cell lines. Many monoterpenes exhibit cytotoxic activity in a variety of tumor cell lines. In most cases, they appear to exert their cytostatic effect by inducing oxidative stress-caused apoptosis, blocking the cell growth cycle, and inhibiting cell invasion and migration. Hence, special attention should be paid to the anti-tumor bioactivity of these monoterpene molecules [38,39].

This study introduced multivariate analysis methods, such as OPLS-DA, spearman correlation matrix, and random forest, to explore REOs’ activity and screen active compounds. We demonstrated their antioxidant activities and their prospects as natural antioxidant resources. Anti-proliferation experiments indicated that these medicinal plants have a broad application prospect in disease treatment and cancer prevention. However, due to the complexity and variable components of natural products, further confirmatory research is needed to fully explore the active components in REOs. Most importantly, the findings may serve as reference information for natural product applications.

## 4. Materials and Methods

### 4.1. Plant Materials

**Figure 5:**
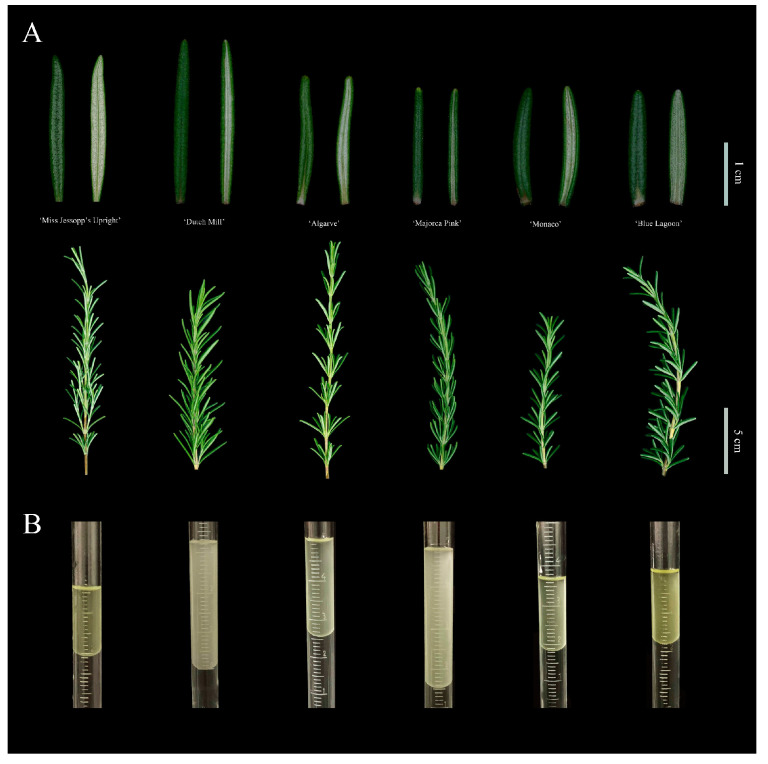
The materials of 6 rosemary cultivars. (**A**) The leaves (adaxial surface and abaxial surface) and branches from 6 rosemary cultivars. (**B**) The REOs extracted from materials.

Six *Rosmarinus officinalis* L. cultivars: *R. officinalis* ‘Miss Jessopp’s Upright’ (Introduction No: CZ2015190), *R. officinalis* ‘Dutch Mill’ (Introduction No: 879-2012), *R. officinalis* ‘Algarve’ (Introduction No: 518-2016), *R. officinalis* ‘Majorca Pink’ (Introduction No: 533-2016), *R. officinalis* ‘Monaco’ (Introduction No: 567-2014), and *R. officinalis* ‘Blue Lagoon’ (Introduction No: 567-2014) (‘MJU’, ‘DM’, ‘AL’, ‘MP’, ‘MO’, and ‘BL’ in short, respectively) (Figure 5A), were collected on August 2020 from the aromatic garden of the Institute of Botany, Chinese Academy of Sciences in Beijing, China (39°59′28″ N, 116°12′24″ E). The 35–50 cm branches at the front of the shrubs were manually harvested and dried at room temperature in the shade for essential oil extraction.

### 4.2. Essential Oil Extraction

The dried *R. officinalis* samples were hydro-distilled for 3 h using a Clevenger-type apparatus. The REO distillates (Figure 5B) were collected, dried over anhydrous sodium sulfate, filtered through 0.22-μm organic microporous filters, and stored in a sealed brown bottle at 4 °C before use.

### 4.3. GC-MS Analysis

To identify and quantitatively analyze their individual components, the obtained REOs were subjected to an Agilent GC 7890 GC-MS system equipped with an Agilent 7000 QqQ MS detector. In brief, 0.8 μL of the volatile oils in n-hexane with a ratio of 1:10 were injected into the instrument. The separation was achieved on an HP-5 capillary column (30 m × 0.25 mm × 0.25 μm), with helium as the carrier gas at a constant flow rate of 1.0 mL/min. The injector temperature was set at 200 °C; the initial temperature was programmed from 40 °C (hold for 2 min) to 260 °C at 4℃/min and to 310 °C at 60 °C/min (hold for 10 min); the detector temperature was set at 250 °C, and a program of 70 eV for the ionization voltage and 35–500 amu for the scan mass range was set for the mass detector. Each component’s retention time and content were obtained from the area percent of the GC-MS chromatogram. The n-alkane homologous series (n-C7-C40) were analyzed using the same column and conditions to calculate the retention index (RI) based on the equation for relative RI. All analyses were run in triplicate.

The compounds were identified based on the RI from the National Institute for Standard and Technology database (NIST, 2014) and literature data, compared with MS database spectra (NIST, 2014), and compared with authentic chemicals (supplementary materials Figure S1).

### 4.4. DPPH Radical Scavenging Assay

The antioxidant activity of REOs was assessed based on their free radical scavenging effect on 2,2-diphenyl-1-picrylhydrazyl (DPPH) radical using the total antioxidant capacity (DPPH method) kit (CominBio, Suzhou, China), as previously reported by Akowuah et al. [40]. In brief, REOs and the positive control TBHQ were diluted using ethanol to 15 mg/mL. Then, 20 μL of each sample or ethanol (the blank group) was taken to react with 380 μL of DPPH solution at room temperature in the dark for 20 min. Finally, 200 μL of the reaction solution was transferred into 96-well plates, and the absorbance at 515 nm was measured on a plated reader. DPPH radical scavenging ratio (%) was calculated as (OD_blank_ − OD_sample_) × 100%/OD_blank_.

### 4.5. ABTS Radical Scavenging Assay

REO’s antioxidant activity was also determined in vitro by ABTS radical scavenging assay [41] using the total antioxidant capacity (ABTS method) kit (CominBio, Suzhou, China). In brief, 10 μL of REOs was diluted with ethanol to 15 mg/mL (TBHQ as a positive control) and mixed with 190 μL of the reaction solution in a 96-well plate. The absorbance at 734 nm was measured within 10 min. The blank was determined after mixing 10 μL of ethanol with 190 μL of reaction solution. ABTS radical scavenging ratio (%) = (OD_blank_ − OD_sample_) × 100%/OD_blank_.

### 4.6. FRAP Reduction Capacity Assay

REOs’ antioxidant activity can also be evaluated by the ferric reducing antioxidant power (FRAP) assay using the total antioxidant capacity (FRAP method) kit (CominBio, Suzhou, China), as reported previously [42]. In operation, 10 μL of 15 mg/mL REOs diluted with ethanol (TBHQ as positive control) or 10 μL of ethanol was mixed and reacted with 190 μL of FRAP reaction solution for 20 min. The absorbance value at 593 nm was determined, and the total antioxidant capacity of each sample was expressed by comparing it to the standard curve of the antioxidant Trolox. The fitting equation of the standards is *y* = 1.2416*x* + 0.0134, R^2^ = 0.9996, where *x* is the Trolox concentration (μmol/mL) and *y* is ΔOD = OD_sample_ − OD_blank_.

### 4.7. Cell Culture

Human immortalized keratinocyte HaCaT, human oral carcinoma cell line KB, human lung adenocarcinoma cell line A549, human hepatoma cell line HepG2, human gastric epithelial cell line NCI-N87, human pancreatic cancer cell line SW1990, and human malignant melanoma cell line A375 were from Cell Resource Center, IBMS, CAMS/PUMC (Beijing, China) and cultured in DMEM or RPMI1640 media supplemented with 1% penicillin-streptomycin solution and 10% FBS (Gibco, CA, USA) at 37 °C in a humidified atmosphere (≥95%) with 5% CO_2_.

### 4.8. Cytotoxicity Activity

The cytotoxic effect of REOs was evaluated by a Cell Counting Kit-8 (CCK-8) assay (Biosharp, Anhui, China) [43]. Cells (100 μL, 8000–15,000 cells/well) were seeded into 96-well plates to grow for 24 h and incubated with six REOs at different concentrations for 24 h. Subsequently, 10 μL of CCK-8 reagent was added to each well. After incubation for 2 h at 37 °C, the concentration of the reduced form of a water-soluble formazan dye was determined using Tecan’s Infinite M200 Pro NanoQuant microplate reader (Thermo Fisher Scientific, MA, USA) at 450 nm. The cell viability was normalized to cells treated with the vehicle under the same conditions. Each test was performed in triplicate. The results of cytotoxicity were expressed as the half inhibition concentration (IC_50_) values.

### 4.9. ROS Scavenging Capacity Assay

The intracellular ROS levels were examined using the fluorescent probe DCFH-DA method [44,45]. First, DCFH-DA (SolarBio, Beijing, China) was diluted with serum-free media at 10 μmol/L and added to the cells treated with six REOs and vitamin C (positive control). After incubating at 37°C for 20 min, the cells were washed with serum-free medium 3 times to fully remove the DCFH-DA that did not enter the cells. The fluorescence intensity of the cells was detected under the excitation wavelength at 488 nm and the emission wavelength at 525 nm using a FACS Calibur LSRFortessa flow cytometer (BD, New Jersey, NJ, USA). Moreover, the fluorescence of cells was observed under an IX73 fluorescence inverted microscope (Olympus, Tokyo, Japan).

### 4.10. Technology for Order Preference by Similarity to an Ideal Solution (TOPSIS)

The mathematical implementation process of TOPSIS was carried out in the following steps [46].

First, the data were normalized by converting the data matrix to the matrix R = (*r_ij_*)*_m×n_*, where *r_ij_* was obtained through vector normalization.

(1) $$r_{ij}=\frac{r_{ij}}{\sqrt{\sum_{i=1}^{m}x_{ij}^{2}}}$$

Second, the positive and negative ideal indicators were determined. The positive ideal indicator set comprises the best performance values among all objects. In the same way, the negative indicator set consists of the worst performance values among all objects. According to the following equations, the positive ideal condition *A^+^* and negative ideal condition *A^−^* are identified, respectively.

(2) $$A^{+}=\left(r_{1}^{+},\;r_{2}^{+},\cdots ,r_{n}^{+}\right)$$

where $r_{j}^{+}$ = optimal value and $r_{i}^{+}\in \{r_{1j},\;r_{2j},\;\cdots ,\;r_{mj}\}$

(3) $$A^{-}=\left(r_{1}^{-},\;r_{2}^{-},\cdots ,r_{n}^{-}\right)$$

$r_{j}^{-}$ = optimal value and $r_{i}^{-}\in \{r_{1j},\;r_{2j},\;\cdots ,\;r_{mj}\}$

Third, the geometric distance was calculated between the object value and the ideal indicator. The Euclidean distance between the alternative *i* and the $A^{+}$ is denoted as $D_{i}^{+}$, and the Euclidean distance between the alternative *i* and the $A^{-}$ is denoted as $D_{i}^{-}$.

(4) $$D_{i}^{+}=\sqrt{\overset{n}{\underset{j=1}{\sum }}(r_{ij}-{r_{j}^{+})}^{2}}$$

(5) $$D_{i}^{-}=\sqrt{\overset{n}{\underset{j=1}{\sum }}(r_{ij}-{r_{j}^{-})}^{2}}$$

Fourth, the similarity to the worst condition was calculated as follows.

(6) $$C_{i}=\frac{D_{i}^{-}}{D_{i}^{+}+D_{i}^{-}}$$

### 4.11. Statistical Analysis

The data were expressed as the mean ± standard deviation (SD) and compared using unpaired Student’s *t*-test or one-way ANOVA. Differences with *p* ≤ 0.05 were considered significant. Spearman’s correlation matrix analysis was performed using SPSS 26.0 software (IBM, New York, NY, USA). IC_50_ was calculated using GraphPad Prism (San Diego, CA, USA). The models of OPLS-DA were developed by the SIMCA program (v. P+ and 14.1, Umetrics Umeå, Sweden). The random forest regression analysis model was implemented with the “randomForest” package in R 4.1.2.

## 5. Conclusions

To our best knowledge, the selected essential oils are representatives of known rosemary germplasm resources, with differences in main components such as camphor, verbenone, α-pinene, bornyl acetate, and 1,8-cineole. Our study proved that REOs have strong cellular antioxidant activity in scavenging ROS. A TOPSIS-random forest model indicated that ‘DM’ REO possesses a more prominent antioxidant capacity with contributions of some of its components. Moreover, the cytotoxicity test of REOs and their major components on human keratinocyte HaCaT suggested that an REO rich in α-pinene is more cytotoxic, providing a reference for evaluating the safety of REOs’ daily application. Additionally, REOs have certain anti-proliferation abilities on six cancer cell lines, with more potent cytotoxicity to human pancreatic cancer cell line SW1990 and gastric epithelial cell line NCI-N87 than other cancer cell lines. Among these REOs, ‘BL’ and ‘MJU’ REOs show obvious anticancer potential. This work utilized different multivariate analysis methods, including component analysis, the exploration of important active compounds, and overall evaluation, and provided a reference value for assessing natural products.

## Figures and Tables

**Table 1:** The chemical composition identified of *R. officinalis* essential oils from six cultivars.

No.	Compound	RI ^a^	RI ^b^	‘MJU’	‘DM’	‘AL’	‘MP’	‘MO’	‘BL’	Identification
1	α-Thujene ^a^	926	924	0.34 ± 0.29	-	-	-	-	-	MS, RI,
2	α-Pinene ^a^	933	932	42.81 ± 2.52	33.15 ± 1.98	7.51 ± 0.11	18.07 ± 1.28	8.94 ± 0.16	9.17 ± 0.79	MS, RI, AC
3	Camphene ^a^	946	946	6.99 ± 0.26	2.45 ± 0.33	1.59 ± 0.18	4.56 ± 0.19	6.06 ± 0.22	2.52 ± 0.26	MS, RI, AC
4	Thuja-2,4(10)-diene ^a^	952	953	-	0.37 ± 0.02	-	-	-	0.55 ± 0.05	MS, RI
5	β-Pinene ^a^	974	974	0.92 ± 0.20	0.89 ± 0.05	1.19 ± 0.28	0.78 ± 0.02	1.14 ± 0.11	0.29 ± 0.26	MS, RI, AC
6	3-Octanone ^g^	987	986	1.51 ± 0.10	-	-	-	-	-	MS, RI,
7	Myrcene ^a^	992	988	3.35 ± 0.46	0.82 ± 0.06	8.05 ± 0.86	0.30 ± 0.01	2.58 ± 0.04	0.56 ± 0.11	MS, RI, AC
8	3-Carene ^a^	1009	1008	-	-	-	-	0.71 ± 0.08	-	MS, RI
9	α-Terpinene ^a^	1016	1017	0.34 ± 0.30	0.53 ± 0.04	-	-	-	-	MS, RI, AC
10	*p*-Cymene ^a^	1024	1025	2.61 ± 0.22	1.30 ± 0.25	10.51 ± 0.70	2.78 ± 0.27	3.02 ± 0.26	2.16 ± 0.02	MS, RI, AC
11	Limonene ^a^	1028	1030	3.31 ± 0.31	2.61 ± 0.43	7.04 ± 0.40	3.48 ± 0.34	3.12 ± 0.30	2.37 ± 0.44	MS, RI, AC
12	1,8-Cineole ^d^	1031	1032	19.95 ± 0.49	23.48 ± 0.87	21.30 ± 1.07	6.01 ± 0.20	13.22 ± 0.32	4.55 ± 0.66	MS, RI, AC
13	γ-Terpinene ^a^	1059	1060	0.61 ± 0.08	0.74 ± 0.10	-	-	0.24 ± 0.21	-	MS, RI, AC
14	Terpinolene ^a^	1088	1087	0.53 ± 0.04	0.65 ± 0.06	-	-	0.55 ± 0.08	-	MS, RI, AC
15	Linalool ^b^	1101	1099	-	1.58 ± 0.09	2.46 ± 0.18	0.29 ± 0.00	0.67 ± 0.07	0.96 ± 0.10	MS, RI, AC
16	Camphor ^c^	1145	1143	6.72 ± 0.70	2.77 ± 0.18	26.80 ± 0.51	56.04 ± 1.47	31.86 ± 0.82	14.18 ± 0.70	MS, RI, AC
17	Borneol ^b^	1166	1166	2.59 ± 0.12	3.67 ± 0.46	0.69 ± 0.14	0.58 ± 0.10	4.03 ± 0.25	3.24 ± 0.35	MS, RI, AC
18	δ-Terpineol ^b^	1167	1166	-	-	0.45 ± 0.08	-	-	-	MS, RI
19	Isopinocamphone ^c^	1174	1173	-	0.53 ± 0.22	0.45 ± 0.04	-	1.60 ± 0.14	0.78 ± 0.04	MS, RI
20	Terpinen-4-ol ^b^	1177	1174	1.19 ± 0.12	0.66 ± 0.59	1.46 ± 0.06	0.66 ± 0.07	1.04 ± 0.13	0.96 ± 0.05	MS, RI, AC
21	p-Cymen-8-ol ^b^	1187	1184	-	-	0.42 ± 0.02	-	-	-	MS, RI
22	α-Terpineol ^b^	1191	1189	1.71 ± 0.31	2.10 ± 0.27	6.61 ± 0.33	1.33 ± 0.07	1.56 ± 0.07	1.12 ± 0.10	MS, RI, AC
23	Mrytenol ^b^	1198	1195	-	0.40 ± 0.08	-	-	-	0.80 ± 0.04	MS, RI
24	trans-Piperitol ^b^	1205	1205	-	0.53 ± 0.06	-	3.02 ± 0.26	0.83 ± 0.07	1.08 ± 0.14	MS, RI
25	Verbenone ^c^	1211	1206	3.64 ± 0.12	14.79 ± 0.57	3.46 ± 0.37	0.99 ± 0.08	4.93 ± 0.28	39.14 ± 0.55	MS, RI, AC
26	Carveol ^b^	1219	1219	-	-	-	0.45 ± 0.04	-	-	MS, RI
27	Geraniol ^b^	1256	1255	0.90 ± 0.13	3.59 ± 0.20	-	-	-	-	MS, RI, AC
28	Bornyl acetate ^e^	1288	1284	-	1.90 ± 0.10	-	-	13.92 ± 0.61	15.57 ± 1.61	MS, RI, AC
29	β-Caryophyllene ^f^	1421	1420	-	0.51 ± 0.06	-	-	-	-	MS, RI, AC
30	α-Bisabolol ^f^	1686	1684	-	-	-	0.65 ± 0.23	-	-	MS, RI, AC
	Monoterpene hydrocarbons			61.80 ± 0.89	43.51 ± 0.90	35.91 ± 1.39	29.97 ± 2.05	26.35 ± 0.61	17.63 ± 1.70	
	Oxygenated Monoterpenes			36.69 ± 0.79	55.98 ± 0.92	64.09 ± 1.39	70.03 ± 2.05	73.65 ± 0.61	82.37 ± 1.70	
	Sesquiterpenes			0.00	0.51 ± 0.06	0.00	0.00	0.00	0.00	
	Others			1.51 ± 0.10	0.00	0.00	0.00	0.00	0.00	

The numbers are arranged in order of retention time, and values (relative peak area percent) represent averages of three determinations (“-” indicates no detection); RI ^a^: calculated retention index of standard mixture of n-alkanes on HP-5MS capillary column; RI ^b^: retention index of the component on semi-standard non-polar capillary column from NIST and reported literature; MS: identified on the basis of comparison with MS database spectra; AC: identified by comparison with an authentic component. ^a^ Monoterpene hydrocarbon (12), ^b^ Monoterpene alcohol (10), ^c^ Monoterpene ketone (03), ^d^ Monoterpene ether (01), ^e^ Monoterpene ester (01), ^f^ Sesquiterpene (02), ^g^ Miscellaneous (01).

**Table 2:** TOPSIS Analysis Results and Ranking of REOs.

REO	$D_{i}^{+}$	$D_{i}^{-}$	$C_{i}$	Ranking
‘MJU’	0.2789	0.0661	0.1915	5
‘DM’	0.0204	0.3286	0.9416	1
‘AL’	0.2272	0.1492	0.3963	4
‘MP’	0.3256	0.0256	0.0730	6
‘MO’	0.1449	0.2099	0.5917	2
‘BL’	0.1843	0.1826	0.4977	3

## Data Availability

The data that support the findings of this study are available upon reasonable request from the corresponding authors (Y.H.).

## Supplementary Materials

The following supporting information can be downloaded at: https://www.mdpi.com/article/10.3390/molecules28020586/s1, Figure S1: The compounds of rosemary essential oils identified by comparison with authentic components.
