# Supplementary material for: Screening of Rosemary Essential Oils with Different Phytochemicals for Antioxidant Capacity, Keratinocyte Cytotoxicity, and Anti-Proliferative Activity
Source: Molecules. 2023 Jan 6;28(2):586. doi: 10.3390/molecules28020586 (PMC9865278; doi:10.3390/molecules28020586)
Supplement: Supplementary file 1 [file molecules-28-00586-s001.zip › molecules-2112687-supplementary.pdf]

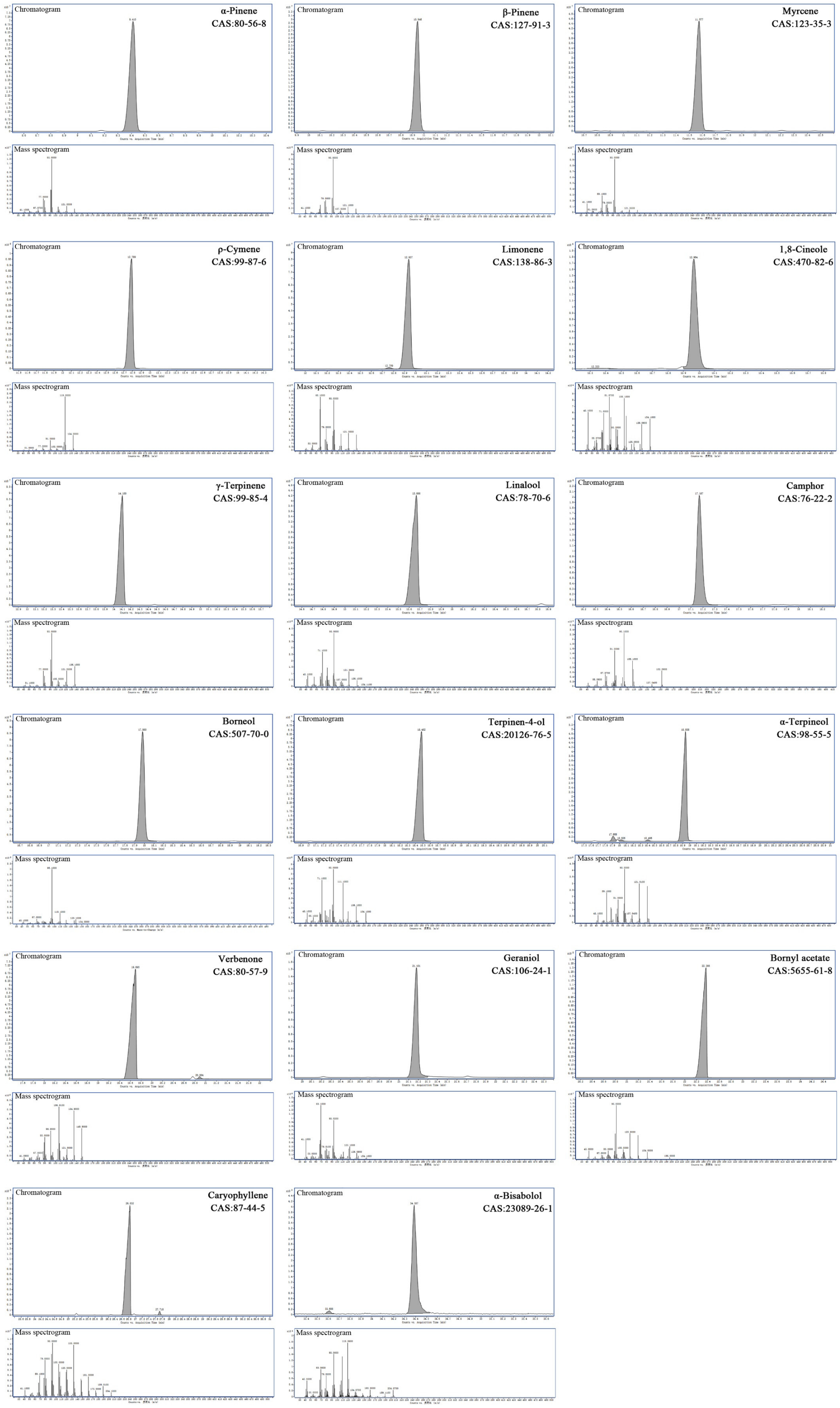

Figure S1. The compounds of rosemary essential oils identified by comparison with authentic components
